# Supplementary material for: Bariatric Surgery Outcomes in Patients with Kidney Transplantation
Source: J Clin Med. 2022 Oct 13;11(20):6030. doi: 10.3390/jcm11206030 (PMC9604744; doi:10.3390/jcm11206030)
Supplement: Supplementary file 1 [file jcm-11-06030-s001.zip › jcm-1880648-supplementary.pdf]

Table S1. Characteristics of the **BS-pre-KTx** at: kidney transplantation, bariatric surgery and 1-/5-year follow-up (n=6).

	**Pre-KTx**	**Pre-BS**	**p-value**	**1 year after BS**	**5 years after BS**	**p-value†**	**p-value††**
**General clinical characteristics**							
Age (years)	44.0 (26.8-47.4)	48.1 (28.6-53.1)	**0.028**				
Weight (Kg)	86.9 (85.0-112.6)	110.7 (104.0-120.0)	**0.043**	79.3 (66.5-94.7)	88.5 (73.0-105.0)	**0.028**	0.249
BMI (Kg/m^2)	37.8 (33.9-38.3)	43.9 (40.6-46.9)	**0.043**	32.9 (26.7-33.7)	33.5 (31.7-38.3)	**0.028**	0116
TWL (%)				26.3 (20.48-28.0)	15.5 (10.1-35.6)		0.116
**Renal function***							
Serum creatinine (mg/dL)	-	1.4 (1.10-1.7)	**-**	1.5 (1.1-1.6)	1.7 (1.6-3.0)	0.500	**0.043**
eGFR mL/min/1.73 m^2	-	45.09 (40.5-62.9)	**-**	47.79 (45.6-58.6)	37.04 (22.2-43.2)	0.500	**0.043**
**Comorbidities**							
Hypertension, n(%)	3 (50.0)	5 (83.3)	0.999	5 (83.3)	3 (50.0)	0.999	0.500
Diabetes, n(%)	0 (0)	1 (16.7)	0.999	1 (16.7)	1 (16.7)	0.999	0.999
Dyslipemia, n(%)	3 (60.0)	5 (83.3)	0.999	3 (50.0)	4 (66.7)	0.625	0.999
**Laboratory characteristics**							
Glucose (mg/dL)	85 (75-99)	94 (86-109)	0.345	84 (77-90)	79 (72-92)	0.459	0.753
Haemoglobin A1c (%)	-	5.4 (5.1-5.7)	-	4.8 (4.8-5.1)	5.3 (4.7-6.5)	0.090	0.465
Total cholesterol, (mg/dL)	201 (176-244)	195 (180-232)	0.999	203 (169-210)	190.5 (186-223)	0.917	0.917
HDL-cholesterol (mg/dL)	-	51 (49-57)	-	55 (41-64)	75 (50-99)	0.586	0.144
LDL-cholesterol (mg/dL)	-	86 (79-143)	-	113 (103-116)	106 (8-128)	0.686	0.715
Triglycerides (mg/dL)	305 (254-337)	204 (158-309)	0.109	102 (84-118)	152 (137-162)	**0.046**	**0.003**

Data are shown as n (%), median (Q1-Q3).

TWL: total weight loss; BMI: body mass index; eGFR: estimated glomerular filtration rate according to CKD-EPI equation.

* n=5 for the comparisons between baseline and 1-year/5-years follow-up as one patient lost the renal graft 13 months after BS.

†*p* values for intra-group comparisons between baseline (prior to BS) and at 1-year follow-up.

†† *p* values for intra-group comparisons between 1-year *vs* 5-year follow-up.

Table S2. Characteristics of the **BS-pre-KTx** at: bariatric surgery (BS), kidney transplantation (KTx), 1-year and 2-year-follow-up (n=5).

	**Pre-BS**	**1 year after BS**	**p-value**	**Pre-KTx**	**2 years after KTx**	**p-value**
**General clinical characteristics**						
Age (years)	38.6 (34.7-61.1)			39.7 (37.9-62.3)		
Weight (Kg)	129.0 (120.0-135.0)	81.0 (80.5-82.0)	0.043	80.0 (79.0-97.0)	84.5 (79.9-90.0)	0.892
BMI (Kg/m^2)	41.7 (39.8-46.9)	26.8 (26.0-27.4)	0.043	27.3 (26.1-30.3)	27.6 (24.8-30.4)	0.893
TWL (%)		40.4 (31.7-41.7)		33.3 (30.6-41.5)	35.3 (29.6-40.8)	0.893
**Comorbidities**						
Hypertension, n(%)	4 (80.0)	1 (20.0)	0.250	2 (40.0)	4 (80.0)	0.500
Diabetes, n(%)	3 (60.0)	2 (40.0)	0.250	2 (40.0)	2 (40.0)	0.999
Dyslipemia, n(%)	4 (80.0)	4 (80.0)	0.999	4 (80.0)	4 (80.0)	0.999
**Laboratory characteristics**						
Glucose (mg/dL)	92 (77-97)	86 (72-88)	0.345	88 (86-96)	92 (86-119)	0.893
Haemoglobin A1c (%)	5.9 (5.8-7.2)	5.55 (4.8-5.8)	0.080	5.40 (5.00-6.30)	5.30 (5.15-6.75)	0.465
Total cholesterol (mg/dL)	182 (133-208)	194 (183-247)	0.500	183 (144-204)	178 (176-192)	0.686
HDL-cholesterol (mg/dL)	39 (35-49)	42 (37-54)	0.343	38 (33-41)	42 (38-47)	0.066
LDL-cholesterol (mg/dL)	79 (63-161)	120 (105-151)	0.715	95 (79-126)	109 (105-139)	0.465
Triglycerides (mg/dL)	163 (122-356)	132 (118-182)	0.225	107 (106-120)	159 (151-172)	0.345

Data are shown as n (%), median (Q1-Q3).

TWL: total weight loss; BMI: body mass index; eGFR: estimated glomerular filtration rate according to CKD-EPI equation.

Figure S1. Eligible candidates for bariatric surgery (BS) 1-year after kidney transplantation (KTx).

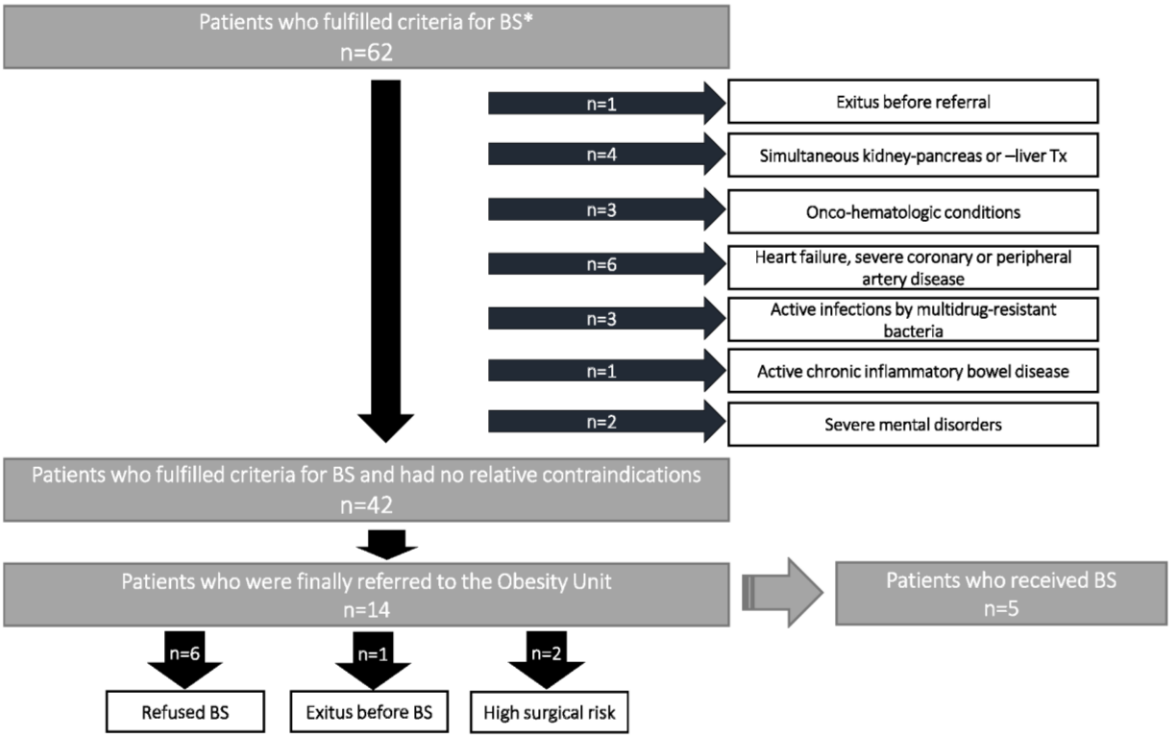

*The indications to undergo bariatric surgery (BS) included: a) BMI ≥35 kg/m2 and 1 or more severe obesity-related comorbidities and b) BMI ≥ 40 kg/m2 and for whom BS would not carry an excessive risk.
